# Mitochondrial Fission Is Required for Angiotensin II-Induced Cardiomyocyte Apoptosis Mediated by a Sirt1-p53 Signaling Pathway

**DOI:** 10.3389/fphar.2018.00176

**Published:** 2018-03-09

**Authors:** Jia Qi, Feng Wang, Ping Yang, Xuelian Wang, Renjie Xu, Jihui Chen, Yanggang Yuan, Zhaoyang Lu, Junli Duan

**Affiliations:** ^1^Department of Pharmacy, Xinhua Hospital, School of Medicine, Shanghai Jiaotong University, Shanghai, China; ^2^Department of Gerontology, Xinhua Hospital, School of Medicine, Shanghai Jiaotong University, Shanghai, China; ^3^Department of Neurology, Shanghai General Hospital, Shanghai Jiaotong University, Shanghai, China; ^4^Department of Nephrology, First Affiliated Hospital of Nanjing Medical University, Nanjing, China

**Keywords:** mitochondrial fission, cardiomyocyte apoptosis, Angiotensin II, hypertension, Drp1

## Abstract

Hypertension-induced cardiac apoptosis is a major contributor to early-stage heart-failure. Our previous studies have found that p53-mediated mitochondrial fission is involved in aldosterone-induced podocyte apoptosis. However, it is not clear that whether p53-induced mitochondrial fission is critical for hypertensive Angiotensin II (AngII)-induced cardiomyocyte apoptosis. In this study, we found that inhibition of the mitochondrial fission protein Drp1 (dynamin-related protein 1) by Mdivi-1 prevented cardiomyocyte apoptosis and cardiac remodeling in SHRs. *In vitro* we found that treatment of cultured neonatal rat cardiomyocytes with AngII induced Drp1 expression, mitochondrial fission, and apoptosis. Knockdown of Drp1 inhibited AngII-induced mitochondrial fission and cardiomyocyte apoptosis. Furthermore, AngII induced p53 acetylation. Knockdown of p53 inhibited AngII-induced Drp1 expression, mitochondrial fission, and cardiomyocyte apoptosis. Besides, we found that Sirt1 was able to reverse AngII-induced p53 acetylation and its binding to the Drp1 promoter, which subsequently inhibited mitochondrial fission and eventually attenuated cardiomyocyte apoptosis. Collectively, these results suggest that AngII degrades Sirt1 to increase p53 acetylation, which induces Drp1 expression and eventually results in cardiomyocyte apoptosis. Sirt1/p53/Drp1dependent mitochondrial fission may be a valuable therapeutic target for hypertension induced heart failure.

## Introduction

Cardiomyocyte apoptosis has been demonstrated to occur in a variety of cardiovasculardiseases. Increased myocyte apoptosis is observed in myocardium from patientswith end-stage heart failureand experimental models of myocardialhypertrophy and failure, including spontaneously hypertensive rats (Liu et al., 2000; Takemura et al., 2013). Many previous studies have reported that Angiotensin II (AngII) is involved in cardiac hypertrophy and apoptosis (Herichova and Szantoova, 2013). Elevation of AngII level is found in hypertensive patients with decompensated hypertrophy and failing hearts, which suggests that AngII stimulation is associated with hypertension induced cardiomyocyte apoptosis (Mehta and Griendling, 2007). However, the detailed mechanisms remain elusive.

Mitochondrial morphology is now recognized as an important determinant of the energetic state of mitochondria. Mitochondria constantly undergo fusion and fission, which are essential for the organelle fidelity. However, abnormal mitochondrial fission is involved in the regulation of apoptosis (Li et al., 2010). Previous studies have reported that mitochondria often fragment into smaller units during apoptosis. Of note, AngII was found to induce apoptosis through Drp1 dependent mitochondrial fission in human umbilical vein endothelial cells (HUVECs) (Chen et al., 2016). However hitherto, whether Drp1 dependent mitochondrial fission participates in the hypertension induced cardiomyocyte apoptosis and the detailed mechanisms underlying Drp1 gene regulation by AngII are still largely unknown.

The tumor suppressor p53 is able to trigger apoptosis via transcription-dependent pathway (Agarwal et al., 1998; Vousden, 2000). Indeed, it has been reported that p53 induced mitochondrial fission by transcriptionally upregulating Drp1 expression in neonatal rat cardiac cells (Li et al., 2010). Sirt1 is a cytoprotective factor involved in the pathogenesis of obesity, diabetes, and aging. Many previous studies have demonstrated that the loss of Sirt1 induced mitochondrial abnormality and dilated cardiomyopathy (Planavila et al., 2012). Importantly, recent studies reported that AngII reduces Sirt1 expression, whereas Sirt1 activation reverses AngII induced cell hypertrophy (Li et al., 2011; Huang et al., 2014). Moreover, Sirt1 is implicated to deacetylate p53 and attenuate the expressions of its downstream target genes including Drp1 in hypoxic cardiac myocytes (Zhang et al., 2011; Aibin Tao et al., 2016). But whether the regulation of Sirt1 on Drp1 and mitochondrial fission participates in AngII induced apoptosis remains to be investigated.

In summary, the clinical and experimental evidences suggest the important role of mitochondrial dynamics in apoptosis. The present study was to investigate whether mitochondrial fission participates in hypertension induced cardiomyocyte apoptosis, and if so to determine the mechanism(s) involved.

## Materials and methods

### Animals and treatments

Male spontaneously hypertensive rats (SHRs) and Wistar-Kyoto rats (WKYs) were obtained from the Animal center, Shanghai Jiaotong University, China. The rats were housed at 25°C with a 12 h light/dark cycle. The animals (14 weeks, 200–240 g) with similar body weight were arranged into five groups (8 rats each) as follows: WKY rats, SHRs, AngII receptor blockers (ARB) treated SHRs, DMSO (the vehicle for Mdivi-1) treated SHR and Mdivi-1 (a chemical compound mitochondrial division inhibitor-1) treated SHRs. The ARB irbesartan (40 mg/kg/day) was placed in drinking water. Mdivi-1 was given as intraperitoneal injection at the dose of 25 mg/kg every other day for 8 weeks. These doses were based on the previous studies (Preau et al., 2016).

The experiments followed the guide for the care and use of Laboratory animals (NIH publication No.85-23, revised 1996) and were under a protocol approved by the Animal care and use committee of Shanghai Jiaotong University.

### Blood pressure measurements

Blood pressure was determined in conscious rats by a tail-cuff method (Kent Scientific Corporation, USA) after 8 weeks of treatment.

### Echocardiographic study

Before the rats were sacrificed, each rat was anesthetized with 2% pentobarbital sodium (50 mg/kg, ip.). After the chests of the rats were shaved, a layer of acoustic coupling gel was applied to the thorax. Echocardiographic evaluation was performed with an echocardiography machine (VisualSonics, Canada). Data were measured by one experienced investigator blinded to the treatment groups. All measurements obtained were the mean of five cardiac cycles on the M-mode tracings.

### TUNEL assay in heart tissue

To detect apoptosis, TUNEL assay were performed with *in situ* Cell Death Detection kit (TUNEL, Roche) according to the manufacturer's instructions. The number of TUNEL-positive cardiomyocytes was counted in 10 randomly selected fields for each animal. The percentage of apoptotic cells was determined by the ratio of apoptotic cells to total cells.

### Electron microscopy

Heart Sample preparations and electron microscopy were performed as described before (Ong et al., 2010). In brief, hearts excised from rats were fixed overnight followed by a 2 mm transverse slice, 3 mm from the apex, was obtained from each heart. Ultrathin sections were examined with a JEOL JEM-1230 transmission electron microscope. Mitochondrial aspect ratio (the ratio of length/width) was quantified by ImageJ as described previously (Song et al., 2015).

### Cell culture

Primary cultures of neonatal rat cardiomyocytes were isolated as described previously (Li et al., 2010). Briefly, hearts were washed and minced in HEPES-buffered saline solution. Tissues were then dispersed in aseries of incubations at 37°C in HEPES-buffered saline solution containing 1.2 mg/ml pancreatin and 0.14 mg/ml collagenase. Supernatants were then collected and centrifuged at 200 g for 5 min. After centrifugation, cells were re-suspended in Dulbecco's modified Eagle medium/F-12 (Gibco) containing 5% heat-inactivated horse serum, 0.1 mM ascorbate, insulin-transferring sodiumselenite media supplement (Sigma, St. Louis, MO, USA), 100 U/ml penicillin, 100 mg/ml streptomycin, and 0.1 mM bromodeoxyuridine. The dissociated cells were pre-plated at 37°C for 1 h. The cells were then diluted to 1 × 10^6^ cells ml/l and plated in 10 mg/ml laminin-coated different culture dishes according to the specific experimental requirements.

### TUNEL assay in cultured cardiomyocytes

After treatment, cardiomyocyte apoptosis was detected by TUNEL assay with an *In Situ* Cell Death Detection Kit (Roche Molecular Biochemicals, Mannheim, Germany) according to the manufacturer's instructions. The number of TUNEL-positive cardiomyocytes was examined using a confocal microscopy.

### Adenoviral construct cloning, packaging, and viral infection

Adenovirus harboring p53 RNA interference (Adp53RNAi), Drp1 RNA interference (AdDrp1RNAi), Sirt1 (AdSirt1), and their scrambled forms were generated and amplified as previously described with the help from HanbioBiotech Co., Ltd. (Shanghai, China). Cardiomyocytes were infected with the virus at the indicated MOI. Infection efficiency was confirmed by Western blotting.

### Mitochondrial staining

For visualization of mitochondria, cardiomyocytes were stained with 50 nM MitoTracker Red. In brief, cells were plated onto coverslips coated with 0.01% poly-L-lysine. After treatment, they were stained for 20 min with 50 nM MitoTracker Red (Molecular Probes) at 37°C before confocal microscope analysis. For quantitation, individual cells were scored as having a fragmented mitochondrial network when >50% of mitochondria in the image were <1 μm in length. At least 100 randomly selected cells were analyzed for each sample.

### Western-blotting

Total protein cell lysates were isolated from cells or from rat hearts as described previously. Protein samples (30 μg per lane) were subjected to immunoblotting analysis with antibodies against acty-p53 (1:1000), Sirt1 (1:1000), Drp1 (1:1000), p53 (1:1000), Bax (1:1000), Bcl2 (1:1000), and GAPDH (1:1000). The blots were visualized with ECL Detection Systems (Amersham, Buckinghamshire, UK). The intensity of the bands was measured using Quantity One Software (Bio-Rad).

### Quantitative real-time PCR

Total RNA extracted from cells or heart tissues with TRIZOL was used to perform the reverse transcription with High Capacity cDNA Archive Kit (Bio-Rad, USA). Real-time quantitative PCR analysis for Drp1 was performed using TaqMan gene expression assays and the 2^−ΔΔCt^ method with housekeeping gene 18S as the endogenous control. The following primers were used for the quantitative RT-PCR: Drp1 Forward: 5′-AGC TGC AAG ACG TCT TCA AC-3′; Reverse: 5′-CAT TCT TCT GCT TCA ACT CC-3′; 18S Forward: 5′-TCA AGA ACG AAA GTC GGA GG-3′; Reverse: 5′-GGA CAT CTA AGG GCA TCA C-3′.

### ChIP analysis

Chromatin immunoprecipitation (ChIP) was carried out with a ChIP kit (Millipore, Billerica, MA) according to manufacturer's instructions. Briefly, chromatin was cross-linked by formaldehyde for 10 min at room temperature. Crosslinking reaction was quenched by glycinesolution for 5 min. Cross-linked chromatin was sonicated into fragments at 4°C. The purified chromatin was immunoprecipitated with p53 antibody (Santa Cruz Biotechnology) or normal rabbit IgG (Millipore). DNA/Protein complexes were collected by the protein G-agarose beads, which were washed and bound DNA was eluted. The eluted DNA was used in PCR amplification with the primers which encompass p53 binding site (BS)1 or BS2 of the rat Drp1 promoter. The primers were as follows: BS1 (corresponding to a 225 bp fragment), Forward: 5′-GGC TGT ATG TGT TCC ATT AC-3′; Reverse: 5′-AGA CAG AAG AGA GTA GGC TC-3′. BS2 (corresponding to a 220 bp fragment), Forward: 5′-AGT AAA GCC TGT CTT GTG TG-39; Reverse: 5′-AAA TAA TCA CAA TAT ACT G-3′.

### Measurement of malondialdehyde

Malondialdehyde (MDA) levels were measured by an ELISA kit (Mybiosource, San Diego, CA) as previous described (Qi et al., 2015). Briefly, the assay sample and buffer were incubated together with MDA-HRP conjugate for 1 h. Then the wells were washed and incubated with a substrate for HRP enzyme. After the stop solution was added, the intensity of color was measured spectrophotometrically at 450 nm. The intensity of the color is inversely proportional to the MDA concentration since MDA and MDA-HRP conjugate compete for the anti-MDA antibody binding site.

### Statistical analysis

Data are presented as the mean ± S.E.M. of at least three independent experiments. We compared parameters between two groups with Student's *t*-test and one-way analysis of variance for multiple comparisons. A value of *p* < 0.05 was considered significant.

## Results

### Mitochondrial fission mediates cardiomyocyte apoptosis in SHR

**Figure 1:**
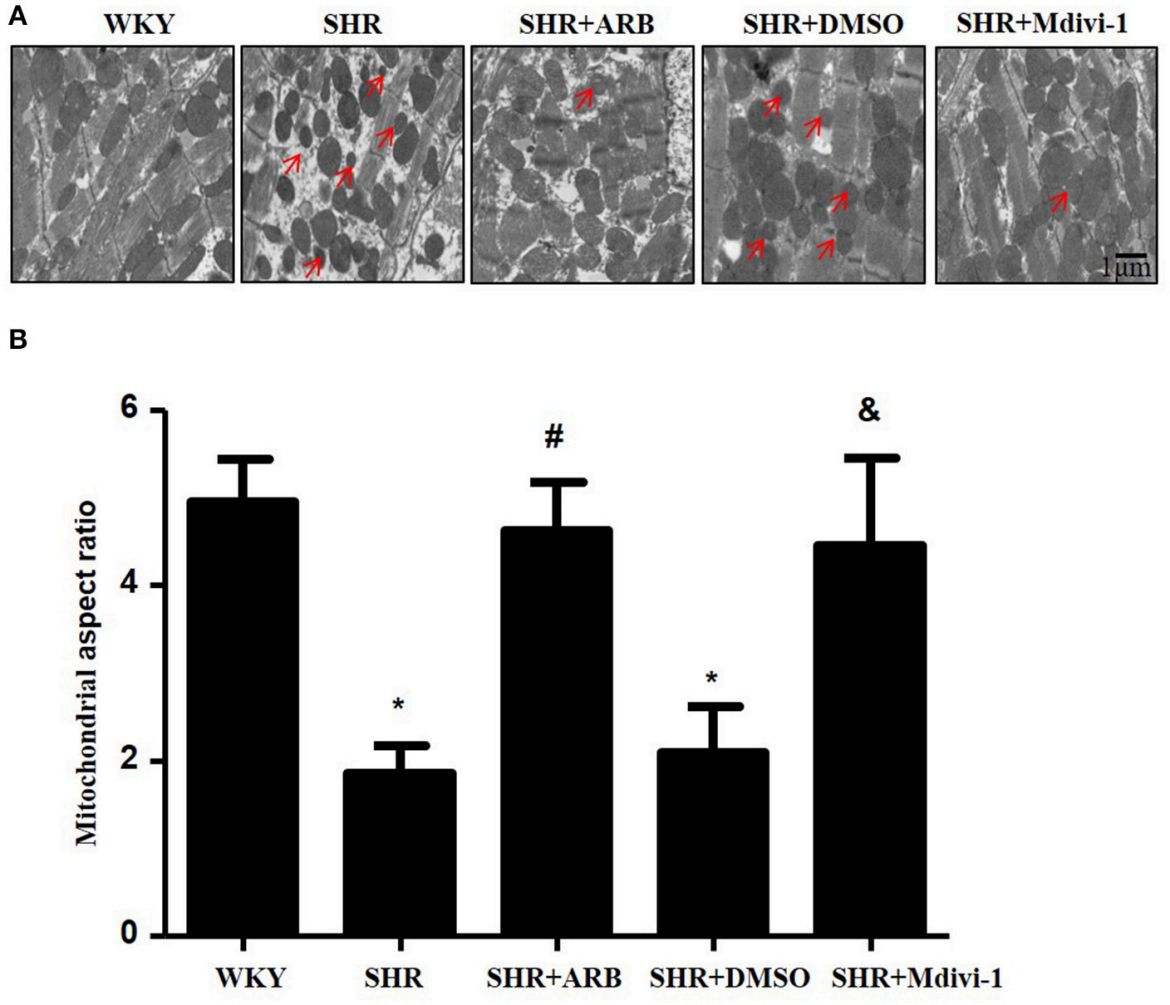
Effect of Mdivi-1 and ARB on mitochondrial fission in SHRs **(A)** Representative EM images depicting relatively fragmented mitochondria (marked with arrow) from respective group. Scale bar, 1 μm. **(B)** Quantitative analysis of the fragmented mitochondria. Values are means ± SEM (*n* = 3 for each group). ^*^p < 0.05 vs. WKY group; #*p* < 0.05 vs. SHRs treated with vehicle; &*p* < 0.05 vs.SHRs treated with DMSO.

To determine whether hypertension induced mitochondrial fission, we performed experiments with SHRs. As shown in Figure 1A, the electron microscopy showed that mitochondrial fission was significantly increased in SHRs compared with WKY group, while treatment with Mdivi-1 abolished the effect in hearts from SHRs. To confirm the effects of AngII on mitochondrial fission, we investigated the effect of ARB on the mitochondrial fission in hearts tissue from SHRs. We observed increased mitochondrial fission in SHRs heart tissue, which was mitigated by ARB (Figures 1A,B).

**Figure 2:**
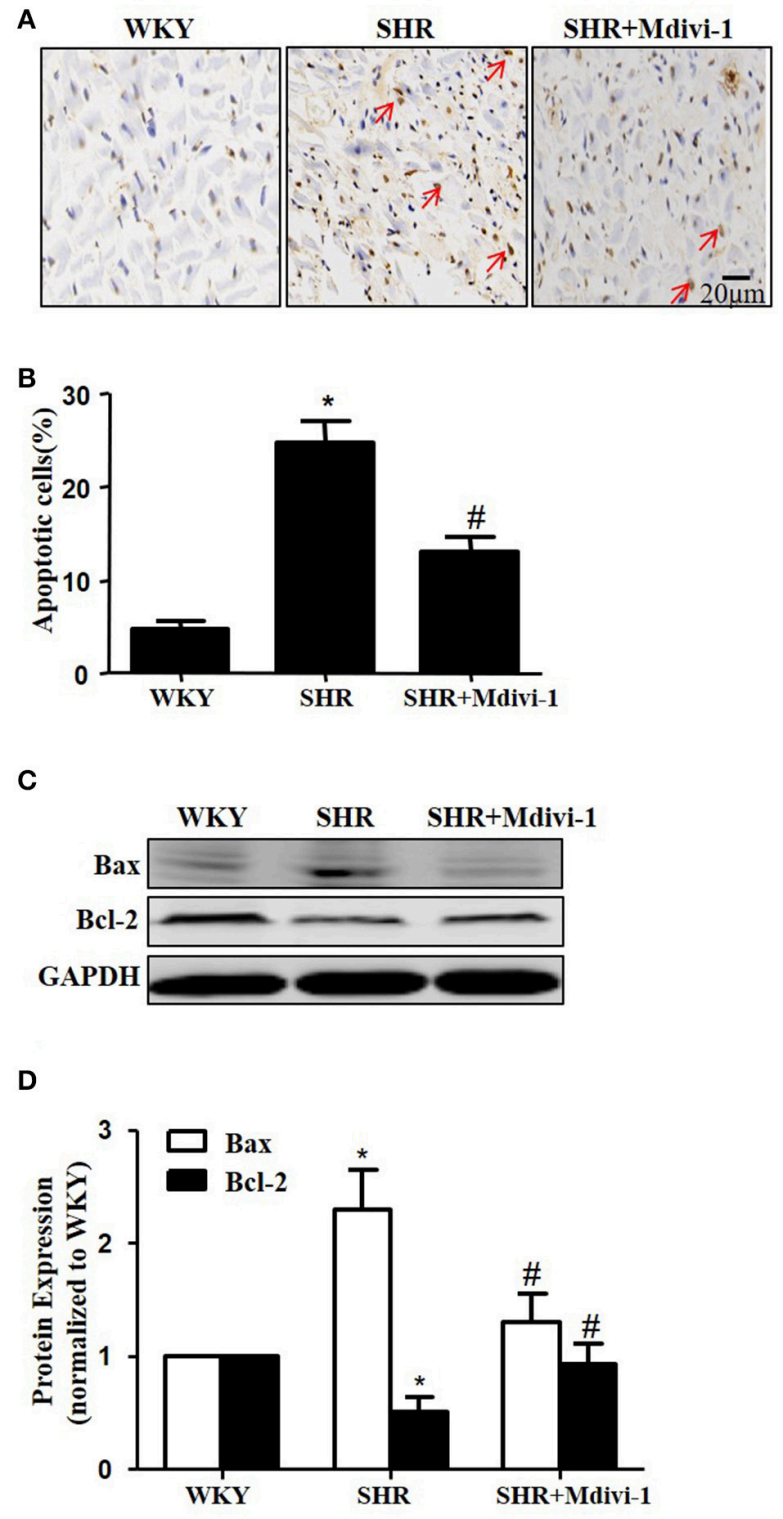
Effect of Mdivi-1 on cardiomyocyte apoptosis in SHRs **(A)** Representative photomicrographs demonstrating TUNEL staining of heart sections from respective group; TUNEL positive cells are brown and indicated by arrows; scale bar, 20 μm; **(B)** Quantitative analysis of TUNEL-positive cells; **(C)** Representative immunoblot images showing the expression of Bax and Bcl-2; **(D)** Quantitative analysis for C. Values are means ± SEM (*n* = 3 for each group). ^*^p < 0.05 vs. WKY group; #*p* < 0.05 vs. SHRs treated with vehicle.

To examine the role of mitochondrial fission on cardiomyocyte apoptosis in hypertensive heart disease, we detected apoptotic cardiomyocytes by TUNEL staining. As shown in Figure 2A, TUNEL-positive cardiomyocyetes per cross-section were increased in SHRs compared with the WKY group, and this induction of apoptosis was significantly reversed by Mdivi-1 treatment (Figures 2A,B). Western blot analysis showed that the ratio of Bax/Bcl-2 decreased in the Mdivi-1treatment group compared with that in the SHRs(treated with vehicle)group (Figures 2C,D). These data indicated that the mitochondrial fission mediated cardiomyocyte apoptosis in hypertensive heart disease.

### Mdivi-1 improves cardiac remodeling in SHRs

**Figure 3:**
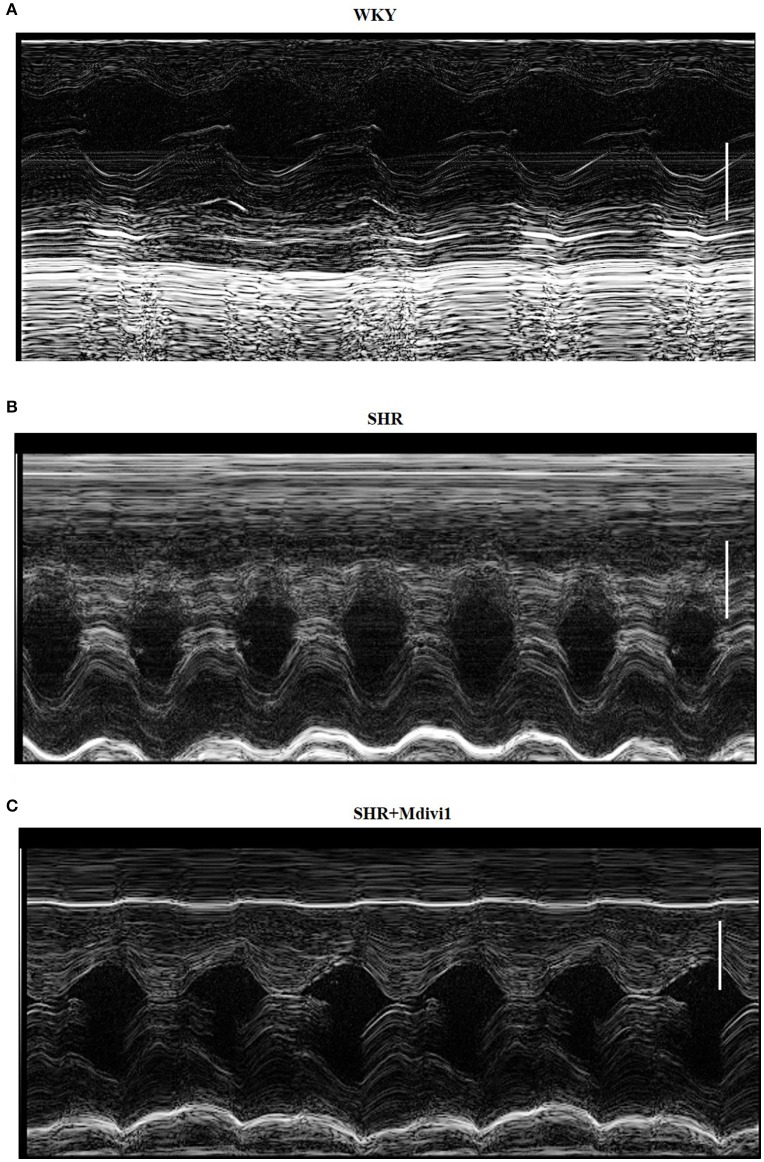
Illustrative left ventricle M-mode echocardiograms. Echocardiographic images from groups: **(A)** WKY; **(B)** SHRs treated with vehicle; **(C)** SHRs treated with Mdivi-1. Scale bar, 3 mm.

To evaluate the effects of Mdivi1 on cardiac function *in vivo*, we performed the echocardiography in rats. Echocardiogram performed before the experiment did not show any differences between groups (data not shown). After 8 weeks, the thickness of the posterior wall was increased in the SHRs compared with those in the WKY group, while Mdivi-1 significantly attenuated this remodeling (*p* < 0.05; Figure 3 and Table 1). However, no significant difference in the LV systolic functions (EF and FS), were observed among all the groups.

**Table 1:** Effect of Mdivi-1 on cardiac remodeling in SHRs.

	**HR(BPM)**	**EF(%)**	**FS(%)**	**LVPW(mm)**	**LVIDd(mm)**	**LVIDs(mm)**
WKY	383 ± 45	85.5 ± 7.41	49.8 ± 5.62	1.72 ± 0.15	5.95 ± 0.85	3.11 ± 0.49
SHR	479 ± 52^*^	87.9 ± 10.1	51.3 ± 7.11	3.02 ± 0.48^*^	6.13 ± 1.14	2.98 ± 0.38
SHR+ Mdivi1	465 ± 47^*^	89.7 ± 12.6	50.6 ± 6.41	2.4 ± 0.19^#^	6.19 ± 1.81	3.18 ± 0.29

*WKY, normotensiveage-matched control rats; SHR, SHR age-matched control rats treated with vehicle; SHR+Mdivi-1, SHR treated with Mdivi-1; HR, heart rate; EF, ejection fraction; FS, fractional shortening; LVPW, left ventricular posterior wall diastolic thickness; LVIDd, diastolic left ventricular internal dimension; LVIDs, systolic left ventricular internal dimension. Data are presented as the mean ± standard error of the mean*.

* *p < 0.05 vs. the WKY group*.

# *p < 0.05 vs. SHR treated with vehicle. n = 8*.

### Drp1 participates in AngII induced mitochondrial fission and apoptosis in cardiomyocytes

**Figure 4:**
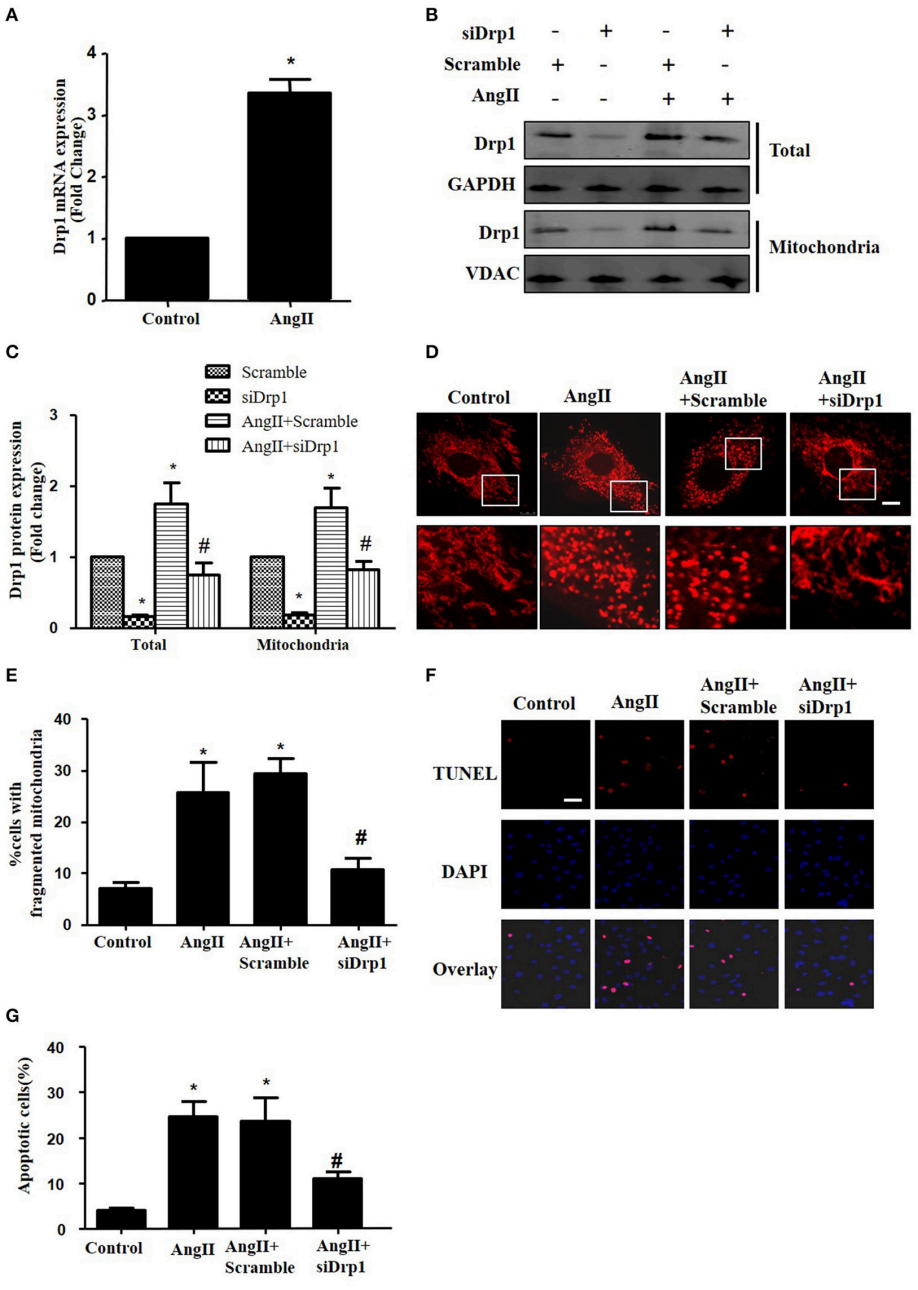
Drp1 participates in AngII induced mitochondrial fission and apoptosis in cardiomyocytes **(A)** AngII induced an increase in Drp1 mRNA expression. Cardiomyocytes were incubated with 1 μM AngII for 24 h. Drp1 mRNA expression were examined by Real-time PCR. **(B)** Cardiomyocytes were infected with either Adscramble(Scramble) or AdsiDrp1 (siDrp1). At 24 h after transfection, cardiomyocytes were stimulated with AngII (1 μmol/L) for 24 h and the expression of total Drp1 and mitochonrial Drp1 protein expression was measured by Western blot analysis. **(C)** Quantitative analysis for **(B)**. **(D,E)** AngII induced mitochondrial fission and apoptosis is Drp1 dependent. Cardiomyocytes were infected with AdsiDrp1 for 24 h and then treated with AngII (1 μM) for 24 h. Micrographs of mitochondrial morphology were visualized by MitoTracker Red staining. Scale bar = 25 μm **(D)**.The cells with fragmented mitochondria were quantified **(E)**. Apoptosis were analyzed by TUNEL assay **(F)**. The apoptotic cells were quantified **(G)**. Values are means ± SEM from three independent experiments. ^*^p < 0.05 vs. Scramble without AngII treatment or control; #*p* < 0.05 vs. scramble plus AngII treatment).

Most recently, AngII was reported to up-regulate the protein expression of Drp1 and induce mitochondrial fission in HUVECs (Chen et al., 2016). To investigate the morphological changes during cardiomyocyte apoptosis triggered by AngII, cultured cardiomyocytes were treated with AngII (1 μM, 24 h) and stained with Mitotracker Red (50 nM, 20 min). As shown in Figures 4A,B, AngII increased mRNA and protein levels of Drp1 in cardiomyocytes. Moreover, the Drp1 in mitochondria was also increased by AngII (Figure 4B). To examine the effect of Drp1 on mitochondrial fission, we downregulated Drp1 expression in cardiomyocytes by siRNAs. As shown in Figures 4B,C, significant reductions in protein expression of Drp1 were observed in cultured cardiomyocytes infected with AdsiDrp1.We further found that Drp1 siRNA prevented AngII induced changes in mitochondrial morphology (Figures 4D,E).

In order to determine the role of mitochondrial fission on AngII-induced cardiomyocyte apoptosis, we next studied the effect of Drp1 inhibition on the cardiomyocyte apoptosis. As shown in Figures 4F,G, TUNEL staining showed that down-regulation of Drp1 by siRNA inhibited AngII-induced cardiomyocyte apoptosis. These data suggested that the Drp1 dependent mitochondrial fission mediates AngII-induced cardiomyocyte apoptosis *in vitro*.

### p53 is involved in AngII-induced mitochondrial fission

**Figure 5:**
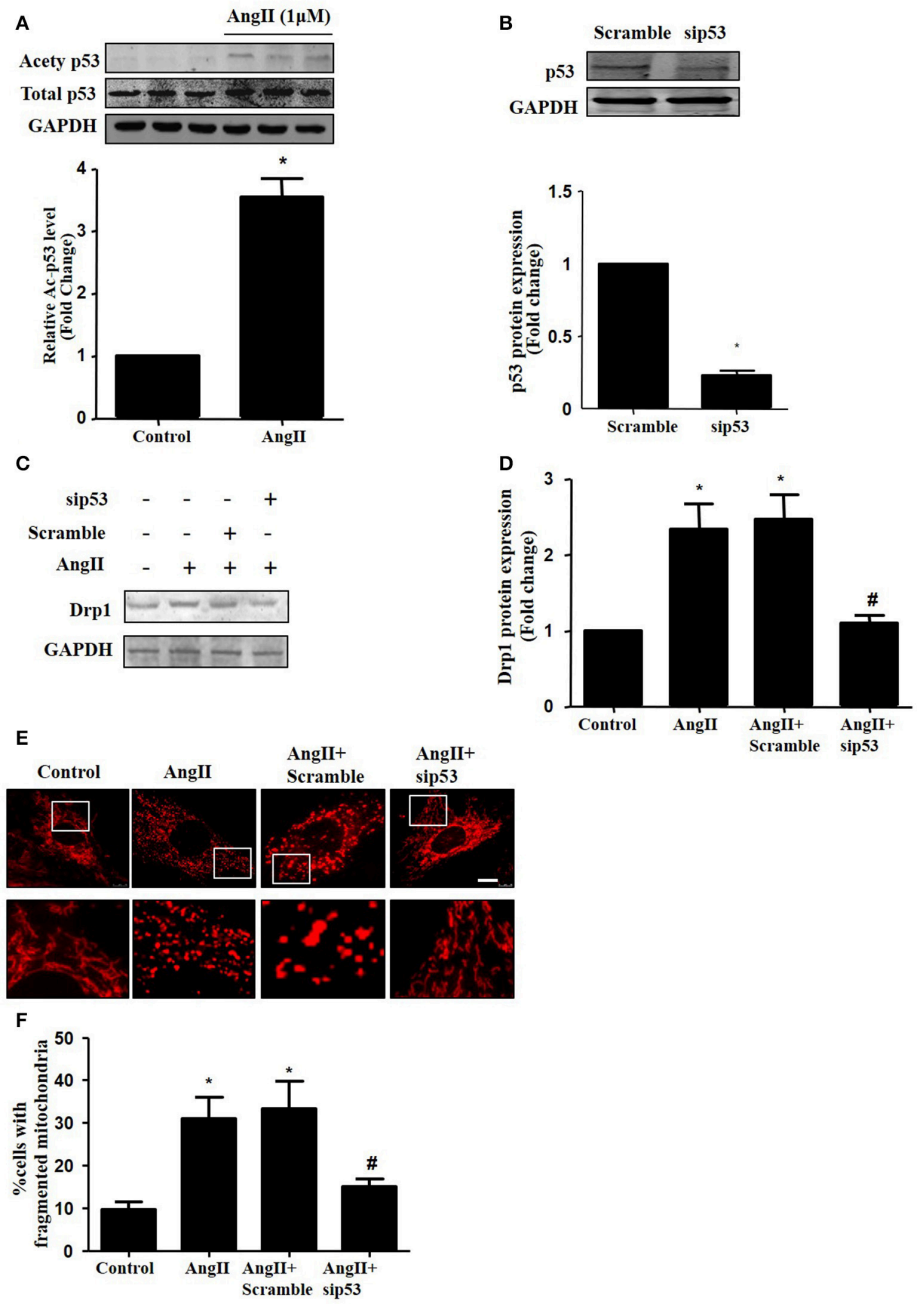
Effect of p53 siRNA on AngII-induced mitochondrial fission. **(A)** Cardiomyocytes were incubated with 1 μM AngII for 24 h. Ace-p53 level was determined by Western-blot. **(B)** Representative immunoblot images showing p53 was successfully inhibited by p53 RNAi. **(C–E)** Effect of sip53 on AngII induced Drp1 expression and mitochondrial fission. Cardiomyocytes were infected with AdsiDrp1 or treated with Mdivi for 24 h and then treated with AngII (1 μM) for 24 h. Drp1 protein expression was analyzed by western blotting **(C)**. Quantitative analysis for C **(D)**. Micrographs of mitochondrial morphology were visualized by MitoTracker Red staining **(E)**. The cells with fragmented mitochondria were quantified **(F)**.Values are means ± SEM (*n* = 3). ^*^p < 0.05 vs. control; #*p* < 0.05 vs. AngII treated cells.

As p53 was reported to induce mitochondrial fission by transcriptionally regulating Drp1 (Yuan et al., 2017), we next tested whether p53 mediated AngII-induced drp1 dependent mitochondrial fission in cardiomyocytes. We found that AngII increased p53 protein acetylation (Figure 5A), which is consistent with previous study (Gao et al., 2014). Next, we employed p53 siRNA to knockdown p53 (Figure 5B). p53 inhibition was able to reduce AngII induced Drp1 expression and subsequent mitochondrial fission (Figures 5C–F). As previous studies have reported p53 over-expression is able to up-regulate Drp1 and induce mitochondrial fission (Li et al., 2010), it seems that endogenous p53 is involved in mediating mitochondrial fission induced by AngII.

### Sirt1 regulates Drp1 expression and mitochondrial fission by deacetylating p53

**Figure 6:**
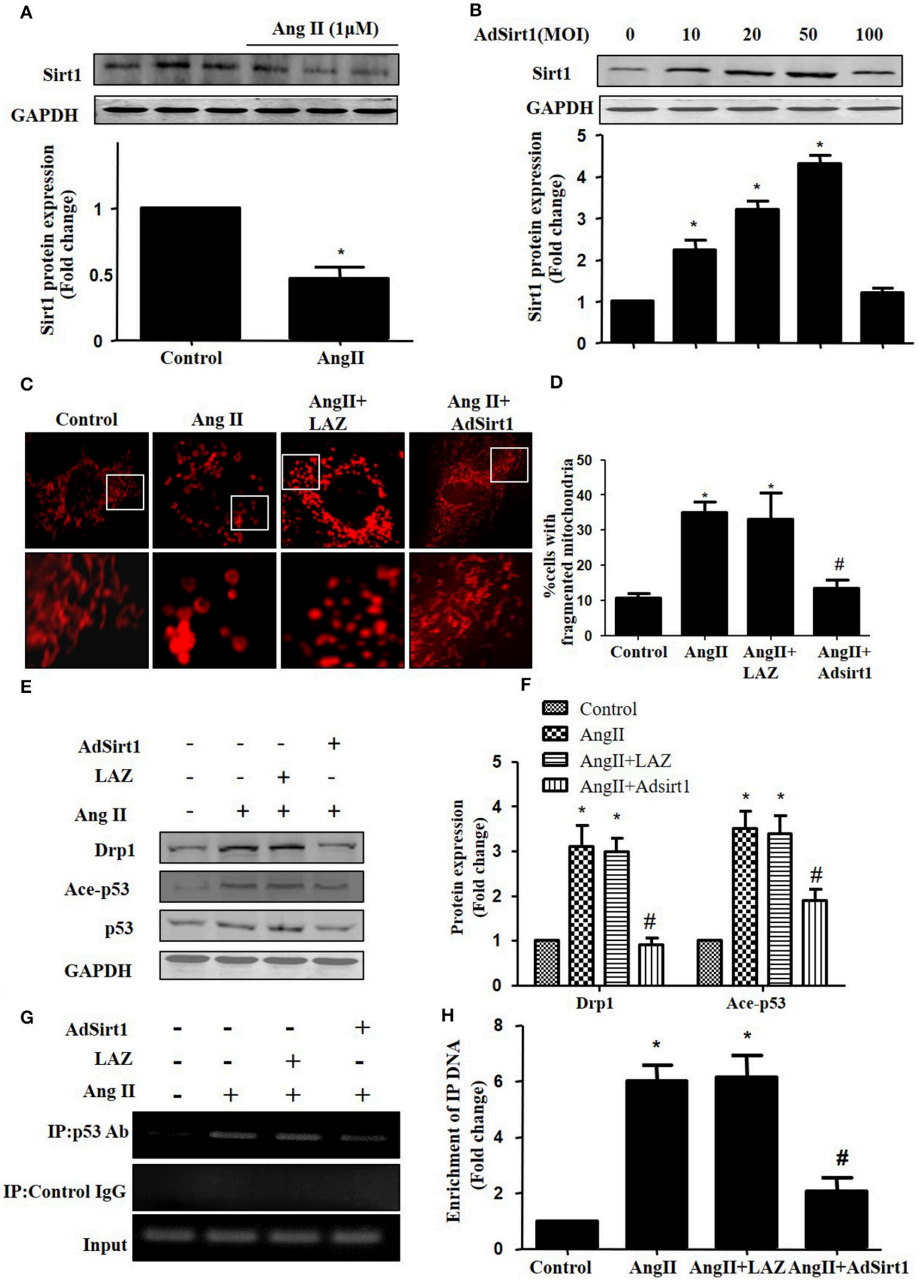
Sirt1 regulates Drp1 expression and mitochondrial fission by deacetylating p53. **(A)** Cardiomyocytes were incubated with 1 μM AngII for 24 h. Sirt1 protein expression was determined by Western-blot. **(B)** AdSirt1 increases Sirt1 expression dose dependently. **(C–H)** Sirt1 inhibited Drp1 expression and mitochondrial fission by deacetylating p53. Cardiomyocytes were transfected with AdSirt1 or LAZ for 24 h and then treated with AngII (1 μM) for 24 h. Micrographs of mitochondrial morphology were visualized by MitoTracker Red staining **(C)**. The cells with fragmented mitochondria were quantified **(D)**. The expression of Sirt1, Ace-p53, p53, and Drp1 were measured by western blot **(E)**. The relative expression of Sirt1, p53, and Drp1 were normalized to control **(F)**.The effects of Sirt1 on the binding of p53 to Drp1 gene promoter (BS2) were detected by chromatin immunoprecipitation (ChIP) analysis **(G)**. The enrichment were quantitively analyzed **(H)**. Values are means ± SEM (*n* = 3). ^*^p < 0.05 vs. control; #*p* < 0.05 vs. AngII treated cells.

Sirt1 has been shown to deacetylate p53 to regulate its DNA-binding activity (Zhang et al., 2011). Thus, we measured Sirt1 expression during AngII treatment. In agreement with previous studies, Sirt1 expression was reduced in AngII treated cardiomyocytes (Figure 6A). Furthermore, we examined whether Sirt1 upregulation was able to deacetylate p53 and inhibit mitochondrial fission in cardiomyocytes. Sirt1 was overexpressed in cardiomyocytes using adenoviral vectors. Figure 6B illustrated that Sirt1 adenovirus (AdSirt1) dose-dependently increased Sirt1 protein expression. Furthermore, Western-blot results indicated that Sirt1 over-expression could deacetylate p53, inhibit Drp1 expression and eventually suppress mitochondrial fission (Figures 6C–H).

As Drp1 is a transcriptional target of p53, we next tested the effect of Sirt1 on p53 binding to the Drp1 promoter in cardiomyocytes by ChIP assay. Although the Drp1 promoter region contains 2 potential p53 DNA binding sites, ChIP assay showed that AngII treatment only led to an elevated binding of p53 to Drp1 promoter (Binding site 2), while Sirt1 over-expression could markedly reduce the effect of AngII on p53 binding to Drp1 promoter(Figures 6F,G).

### Effects of ARB on the expressions of Sirt1, acety-p53, and Drp1 in the hearts from SHRs

**Figure 7:**
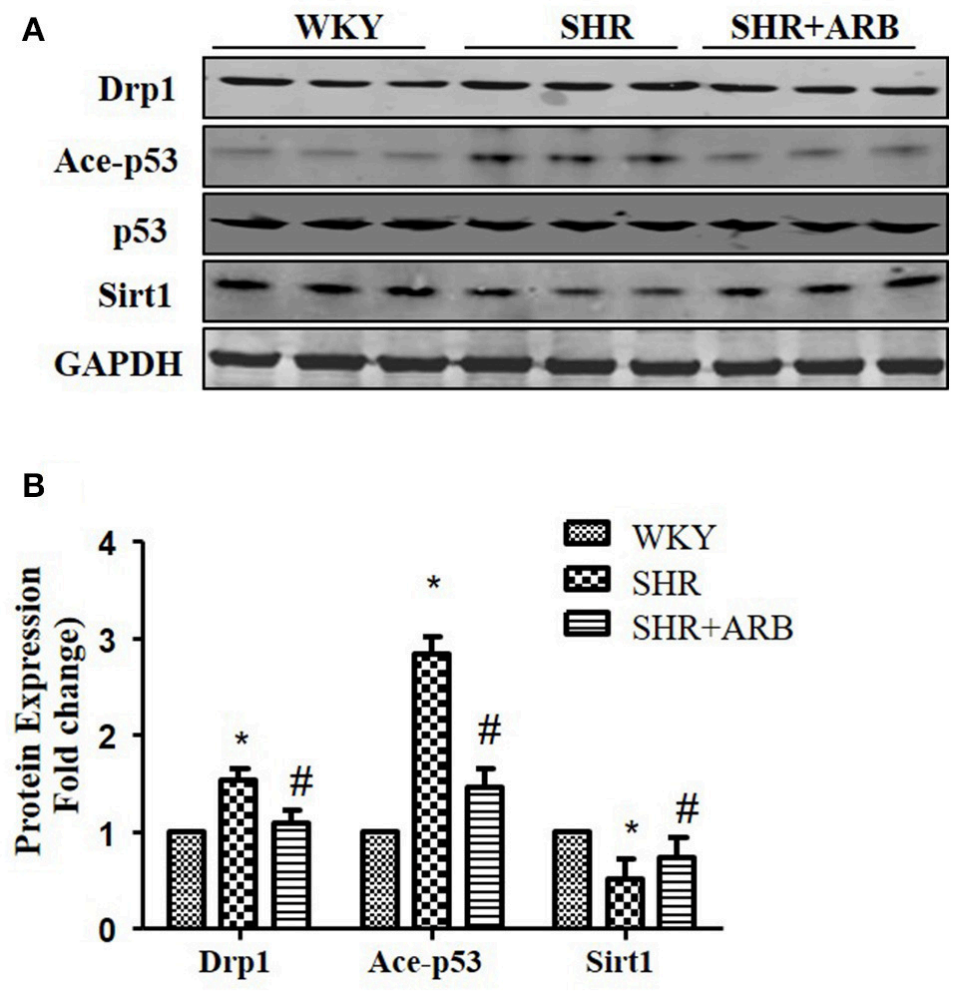
The Effect of ARB on protein expression of Sirt1, Ace-p53, p53, and Drp1 in SHR hearts. **(A)** Reduced Sirt1 expression, increased p53 acetylation, and up-regulation of Drp1 were shown in SHR heart tissue, which were restored by ARB. **(B)** The relative expression of Sirt1, p53, and Drp1 were normalized to the WKY group. ^*^p < 0.05 vs. WKY group; #*p* < 0.05 vs. SHR group.

To confirm Sirt1/p53/Drp1pathway *in vivo*, we next examined the protein expression in the heart tissue of SHRs and SHRs treated with ARB. We observed reduced Sirt1 expression, increased p53 acetylation and up-regulation of Drp1 in SHRs heart tissue. On treatment with ARB, the Sirt1, and acety-p53 level were recovered (Figures 7A,B). Consequently, AngII-induced mitochondrial fission and apoptosis were ameliorated (Figures 1, 2).

## Discussion

Our present work shows that inhibiting mitochondrial fission by Mdivi-1 prevents cardiomyocyte apoptosis and attenuates cardiac remodeling in SHRs. *In vitro* we found that AngII induced mitochondrial fission and apoptosisvia an elaborate mechanism, involving transcriptionally activation of Drp1 through p53 acetylation induced by Sirt1 degradation. Our findings provide a well-characterized mechanism underlying which AngII stimulates mitochondrial fission and consequent apoptosis, which may offer a new therapeutic strategy for treating hypertension-induced heart failure via the Sirt1-p53-Drp1 pathway.

Changes in mitochondrial morphology are key determinants of the initiation of cardiomyocyte apoptosis. Our previous studies have reported that cisplatin led to apoptosis of renal tubular epithelial cells via induction of mitochondrial fission (Zhao et al., 2017). In the heart, some studies haveexamined the role of mitochondrial fission in ischemia/reperfusion injury (Ong et al., 2010; Wang et al., 2011). Herein, we tested whether mitochondrial fission participated in hypertension induced cardiomyocyte apoptosis and cardiac remodeling. We found increased mitochondrial fission and apoptosis in heart tissue from SHRs, which was significantly inhibited by pharmacological Drp1 inhibition by Mdivi-1 or ARB (Figures 1, 2). In addition, mitochondrial fission was accompanied by specific morphological changes in SHRs such as cristae disorganization, which was reversed by ARB, although no statistical significance was observed (Figure S1). These results suggest that mitochondrial fission is required for cardiomyocyte apoptosis in SHRs. Importantly, Mdivi1 also attenuated cardiac remodeling in SHRs (Figure 3 and Table 1). Although no effect of Mdivi1 on EF, FS, and LVID was observed, the LVPW was found to be improved, which suggested the potential clinical relevance. Of note, as the heart rate is different among the groups, the ejection fraction or fractional shortening should be different. However, no significant difference was observed in this study. It is probabaly because of the hypertensive cardiac compensatory. As apoptosis occurs earlier than BP in SHRs, the apoptosis in SHRs is thought to be, at least in part, dependent on AngII (Liu et al., 2000). Our *in vitro* study also found that AngII induced mitochondrial fission and apoptosis in cardiomyocytes. Crucially, Mdivi-1 inhibited AngII induced apoptosis. As Mdivi-1 had no effect on the blood pressure in SHRs (Table S1), this suggested that AngII might contribute to cardiomyocyte apoptosis in SHRs via inducing Drp1 dependent mitochondrial fission independent of blood pressure.

Several studieshave revealed that Drp1 participated in mitochondrial fission and subsequent apoptosis induced by various stimuli (Oettinghaus et al., 2016). For example, Hypoxia could trigger mitochondrial fission through Drp1 in cardiomyocyte (Li et al., 2010). Recently, it has been reported that AngII induced apoptosis by up-regulating Drp1 protein expression in HUVECs (Chen et al., 2016). However, whether Drp1 is essential for AngII induced cardiomyocyte apoptosis and the upstream signals that regulate the expression of Drp1 are still unknown. Our present work demonstrated that up-regulation of Drp1 was involved in AngII induced mitochondrial fission and apoptosis in cardiomyocyte. These results indicated that the apoptotic effect of AngII might be caused by up-regulation of Drp1 signaling cascade and mitochondrial fission. Of note, Drp1-independent mechanism that participates in mitochondrial fission during apoptosis has been reported recently (Martinou and Youle, 2011). The actin cytoskeleton F-actin was found to contribute to non–Drp1-related mechanisms of mitochondrial fission (Li et al., 2015). However, their mechanistic roles in mitochondrial fission are still elusive.

P53 is a well-known tumor suppressor which may initiate the mitochondrial apoptotic pathway. Many studies have indicated that p53 may convey the death signal via multiple mediators. For example, p53 was found to regulate the transcription of pro-apoptotic proteins (Miyashita and Reed, 1995; Sahin and DePinho, 2012). Importantly, several recent studies demonstrated that p53 promoted Drp1-dependent mitochondrial fission via direct transcriptional regulation of Drp1 (Li et al., 2010). In addition, p53 protein hyperacetylation was found to stabilize and activate itself to initiate apoptosis (Appella and Anderson, 2001; Brooks and Gu, 2003). Consistent with the findings of other studies that p53 protein acetylation mediated Doxorubicin-induced cardiomyocyte apoptosis (Zhang et al., 2011), our present study showed that p53 acetylation mediated AngII-induced apoptosis through targeting Drp1-dependent mitochondrial fission. However, as multiple lines of evidences suggest that p53 functions by acting on mitochondria directly (Nithipongvanitch et al., 2007), p53 may also regulate apoptosis independent of its transcription activation.

Sirt1 is a cytoprotective factor implicated in various cellular functions including control of cell cycle and apoptosis. Recent study has found that AngII activated JNK1/2 to reduce Sirt1 levels via ubiquitin degradation (Huang et al., 2014). In addition, Sirt1 is reported to deacetylate p53 and attenuate its ability to transactivate its downstream target genes (Lee and Gu, 2013). Moreover, Sirt1 over-expression rescued mitochondrial density and fission–fusion balance in neurodegeneration models (Reddy, 2014). Our present study found that Sirt1 over-expression reversed AngII-induced p53 acetylation and its binding to the Drp1 promoter, which subsequently inhibited mitochondrial fission and eventually attenuated cardiomyocyte apoptosis. Moreover, it has been reported that in Hela cells Drp1 downregulation leads to mitochondrial dysfunction increasing ROS production (Parone et al., 2008). Present study found that Sirt1 over-expression reversed AngII-induced oxidative stress, which indicates that Sirt1-regulated mitochondrial fission may mediate AngII-induced cardiomyocyte apoptosis through increasing oxidative stress (Figure S3).

Of note, although Drp1 is an essential protein in mitochondrial fission, the morphological changes observed in mithocondrial fusion are dependent on a complex molecular mechanism that involves several proteins. Indeed, increased mitochondrial fragmentation is linked with decrease of the OPA1 expression in failing hearts (Sahin and DePinho, 2012). And MFN2 inhibition leads to mitochondrial depolarization and cell death in cardiomyocytes. Our study showed that the expression levels of MFN2 and OPA1 were decreased in SHRs compared to the levels in WKY rats and ARB-treated SHRs (Figure S2). As both mitochondrial fusion and fission are essential for maintaining mitochondrial quality and function, so further investigation is still needed to confirm whether and how mitochondrial fusion is involved in AngII induced cardiomyocyte apoptosis.

**Figure 8:**
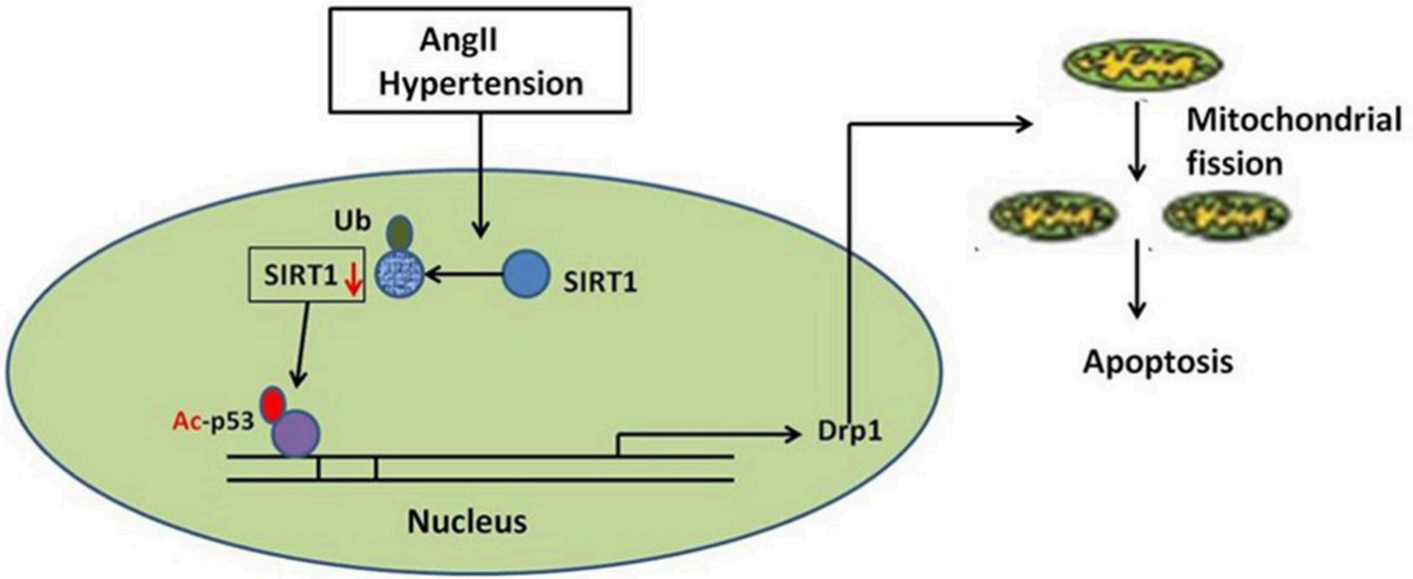
Schematic model of AngII-induced mitochondrial fission and apoptosis in cardiomyocyte. AngII degrades Sirt1, which increases p53 acetylation, resulting in enhanced Drp1 expression, which induces mitochondrial fission and consequent apoptosis in cardiomyocyte.

In summary, our studies identify a novel route for mitochondrial dynamics regulation via Sirt1/p53/Drp1 pathway, which may affect hypertension induced cardiomyocyte apoptosis. AngII degrades Sirt1, which increases p53 acetylation, resulting in enhanced Drp1 expression, which induces mitochondrial fission and consequent apoptosis in cardiomyocyte (Figure 8). Although a deeper evaluation of this mechanism in vivo needs to be done in future, these findings may suggest a strategy for treating hypertension-induced heart failure.

## Author contributions

JQ: performed the experiments, designed study, and analyzed data; FW and YY: performed the experiments and analyzed data; XW: performed the experiments; RX: performed ChIP analysis; PY: cell culture; JC: performed experiments, data analysis; ZL: study design and data analysis, writing the paper; JD: designed entire research.

### Conflict of interest statement

The authors declare that the research was conducted in the absence of any commercial or financial relationships that could be construed as a potential conflict of interest.

## Supplementary material

Figure S1 The effect of ARB on Mitochondrial cristae disorganization. Representative EM images depicting relatively cristae disorganized mitochondria (marked with arrow) from respective group. Scale bar, 1 μm.

Figure S2 The effect of ARB on the expression of Opa1 and Mfn2. Representative immunoblot images showing the expression of Opa1 and Mfn2.

Figure S3 Sirt1 inhibits AngII-induced oxidative stress in cardiomyocytes. Effect of Sirt1 on MDA levels in cells was assessed by ELISA. ^*^*p* < 0.05 vs. Control; #*p* < 0.05 vs. AngIItreated cells. *n* = 3.

Table S1 Effect of Mdivi-1 on blood pressure in SHRs.
