# Supplementary figures and images for: Mitochondrial Fission Is Required for Angiotensin II-Induced Cardiomyocyte Apoptosis Mediated by a Sirt1-p53 Signaling Pathway
Source: Front Pharmacol. 2018 Mar 9;9:176. doi: 10.3389/fphar.2018.00176 (PMC5854948; doi:10.3389/fphar.2018.00176)

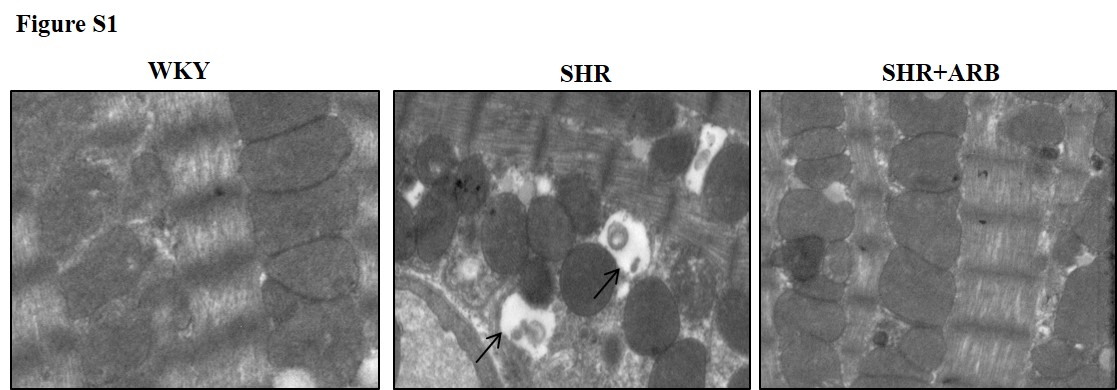

Supplement: Figure S1 — The effect of ARB on Mitochondrial cristae disorganization. Representative EM images depicting relatively cristae disorganized mitochondria (marked with arrow) from respective group. Scale bar, 1 μm. [file Image1.JPEG]

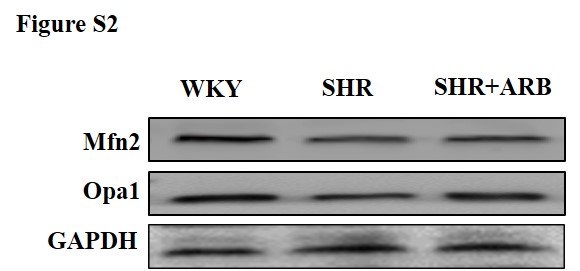

Supplement: Figure S2 — The effect of ARB on the expression of Opa1 and Mfn2. Representative immunoblot images showing the expression of Opa1 and Mfn2. [file Image2.JPEG]

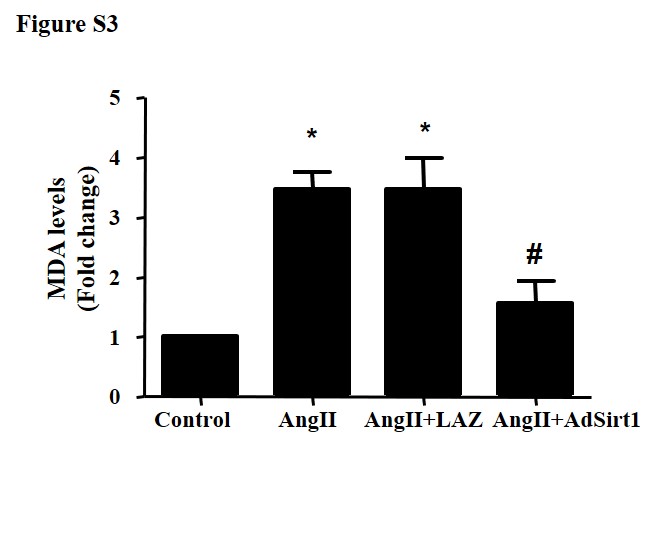

Supplement: Figure S3 — Sirt1 inhibits AngII-induced oxidative stress in cardiomyocytes. Effect of Sirt1 on MDA levels in cells was assessed by ELISA. *p < 0.05 vs. Control; #p < 0.05 vs. AngIItreated cells. n = 3. [file Image3.JPEG]
