# Supplementary material for: Mitochondrial Fission Is Required for Angiotensin II-Induced Cardiomyocyte Apoptosis Mediated by a Sirt1-p53 Signaling Pathway
Source: Front Pharmacol. 2018 Mar 9;9:176. doi: 10.3389/fphar.2018.00176 (PMC5854948; doi:10.3389/fphar.2018.00176)
Supplement: Table S1 — Effect of Mdivi-1 on blood pressure in SHRs. [file Table1.pdf]

Supplemental Material

Supplemental Table S1.

Effect of Mdivi-1 on blood pressure in SHRs.

	WKY	SHR	SHR+Mdivi-1
**SAP**	**122.5±9.2**	**195.2±7.9***	**196.7±5.9***
**DAP**	**103.1±6.1**	**163.1±10.1***	**159.3±6.4***

WKY: normotensiveage-matched control rats, SHR: SHR age-matched control rats treated with vehicle, SHR+Mdivi-1: SHR treated with Mdivi-1. SAP, DAP: systolic and diastolic blood pressure at 22-week-old age. Data are presented as the mean ± standard error of the mean. *p< 0.05 versus the WKY group. n=8.
